# Liver Resection Under Inflow Occlusion: A Bloodless Operation?

**DOI:** 10.1155/1994/48068

**Published:** 1994

**Authors:** Claude Huguet

**Affiliations:** Centre Hospitalier Princesse Grace BP No 480, MC 98082 Monaco Cedex Monaco France


7. Stephen, M. S., RossSheil, A. G., Thompson, J. F. *et al.* (1990) Aortic occlusion and vascular isolation allowing avascular hepatic resection. *Archives of Surgery* **125**, 1482–57
8. Emre, S., Schwartz, M. E., Katz, E., Miller, C. M. (1993) Liver Resection under total vascular isolation. *Annals of Surgery*, **217**, 15–19

O. James Garden
University Department of Surgery
and Scottish Liver Transplant Unit
Royal Infirmary
Edinburgh
EH3 9YW, Scotland
United Kingdom

### LIVER RESECTION UNDER INFLOW OCCLUSION: A BLOODLESS OPERATION?

### ABSTRACT

*Stephen, M. S., Sheil, A. G. R., Thompson, J. F., Wilson, T. and Boland, S. L. (1990) Aortic occlusion and vascular isolation allowing avascular hepatic resection. Archives of Surgery*; **25**: 1482–1485.

• **Occlusion of the supracellac abdominal aorta and hepatic vascular isolation were employed in a series of 15 patients as a definitive method to allow avascular hepatic resection. The series was compared with an earlier group of patients treated conventionally. In the avascular hepatic resection group there was no mortality; hypotenslon did not occur at the time of hepatic vascular isolation; rapid, accurate excision of the hepatic lesions could be achieved in a bloodless field; resection of midline lesions and those involving the great veins was possible; and "segmentectomies," or resections crossing segmental boundaries, could be performed where previously formal hepatic lobectomies were required. Concomitantly, the greatest amount of uninvolved hepatic parenchyma remained in situ. There was increased ease of operative management, reduced blood loss, and reduced operating time (mean, 2.8 hours).**

### PAPER DISCUSSION

KEY WORDS: Liver resection, liver ischaemia, inflow occlusion.

Control of blood loss is the main objective of surgeons during the performance of hepatic resection. Reduction of peroperative haemorrhage appears today as the main factor to avoid operative mortality and to lower post operative morbidity rate[1–3]. Any attempt to achieve bloodless liver resection is thus welcome. The article by Stephen *et al.*, reports an experience of 15 liver resections performed with the use of vascular isolation according to the technique of Heaney [4]. In 1966 this author reported 3 cases of liver resection with combined clamping of the supracoeliac aorta, the portal triad (Pringle manoeuvre) and the inferior vena cava (IVC) below and above the liver at the intra-pericardial level. No further publications have appeared. The technique of vascular isolation applied by the Sydney team is very similar to the Heaney procedure but differs by clamping the supra-hepatic IVC below the diaphragm which prevents blood pooling in the liver via the diaphragmatic veins, and subsequent bleeding during liver transection. Even if this technical modification represents an improvement, the level of clamping of the infrahepatic IVC below the right adrenal vein is not satisfactory for the same reason. Hepatic vascular exclusion is a fairly well tolerated procedure if the IVC exclusion is complete, with interruption of the venous flow coming from the diaphragmatic, right adrenal and lumbar veins. From our experience correct application of the caval clamps is thus most important if good haemodynamic tolerance is to be achieved[5,6].

Clamping of the supracoeliac abdominal aorta has been completely abandoned by us for several reasons: good tolerance of simple triple exclusion (if the technique is adequate), difficulty in reaching the aorta in cases of massive liver tumour which represents the typical indication for hepatic vascular exclusion, and risk of paraplegia secondary to ischaemia of the spinal cord if Adamkiewicz's artery is cross clamped. One has to admit that as approach to the aorta through the diaphragmatic crura results in thoracic rather than abdominal aortic clamping. No adverse effect of aortic occlusion was observed on kidney, gastrointestinal nor spinal cord function in this series, mainly because of the very limited ischemic period (19.5 ± 7 min.). The authors deserve special congratulations for the speedy procedure which illustrates their skill. I am concerned about the example their publication represents, as it might be followed by less experienced surgeons, with a major risk of severe complications.

As far as the comparison with the conventional technique is concerned (10 previous consecutive patients), no definitive conclusion can be drawn from this type of historical data. Nor is it possible to assess whether the 2 groups are similar: 9 hemihepatectomies out of 10 in the conventional group, 9 segmentectomies out of 15 in the avascular group. It is true that blood loss appears to be reduced in the avascular group (1720 ± 800 versus 3940 ± 1600 ml) but I emphasize that haemorrhage remains significant with the proposed technique. And it should be stressed that not a single cirrhotic patient was included in this study.

Finally, I cannot agree with Stephen *et al.*, when they claim that safe and speedy resections are possible without precise regard for the anatomic hepatic segments. Functional viability of the remnant liver presupposes vascular and biliary integrity which is achieved, in our opinion, by precise and unspeedy technique respecting anatomic landmarks. The role of peroperative ultrasonography has to be stressed in this regard.

The good tolerance of the liver to prolonged normothermic ischemia[7], up to 90 minutes in the absence of hepatic dysfunction prior to surgery, is the main argument for performing any liver resection using vascular clamping to reduce haemorrage and avoid any blood transfusion. Depending upon the case, vascular clamping may be a simple Pringle manoeuver, the most common situation, or less frequently complete hepatic vascular exclusion by clamping the hepatic pedicle and the vena cava below and above the liver when the tumor is massive and/or badly located, i.e., close to the hepatic veins or inferior vena cava.

Associated aorta clamping does not offer any further advantage and is associated with its own complications. For this reason, it appears unnecessary in our practice today.

### REFERENCES

1. Delva, E., Camus, Y., Nordlinger, B., Hannoun, L., Parc, R., Deriaz, H., Lienhart, A. and Huguet, C. (1989) Vascular occlusion for liver resections. Operative management and tolerance to hepatic ischemia: 142 cases. *Ann Surg.*, **209**, 211–128
2. Nagasue, N., Yukaya H., Ogawa, Y., Hirose, S. and Okita, M. (1985) Segmental and subsegmental resections of the cirrhotic liver under hepatic inflow and outflow occlusion. *Br. J. Surg.*, **72**, 565–568
3. Bismuth, H., Castaing, D. and Garden, O. J. (1989) Major hepatic resection under total vascular exclusion. *Ann Surg.*, **210**, 13–19
4. Heaney, J. P., Stanton, W. K., Halbert, D. S., Seidel, J. and Vice, T. (1966) An improved technique for vascular isolation to the liver experimental study and case reports. *Ann Surg.*, **163**, 237–241
5. Huguet, C., Addario-Chieco, P., Gavelli, A., Arrigo, E., Harb, J. and Roger-Clement, R. (1992) Technique of hepatic vascular exclusion for extensive liver resection. *Am. J. Surg.*, **163**, 602–605.
6. Huguet, C., Nordlinger, B., Galopin, J. J., Bloch, P. and Gallot, D. (1987) Normothermic hepatic vascular exclusion for extensive hepatectomy. *Int. Surg.*, **72**, 78–81
7. Huguet, C., Gavelli, A., Addario-Chieco, P., Bona, S., Harb, J., Joseph, J. M., Jobard, J., Gramaglia, M. and Lasserre, M. (1992) Liver ischemia for hepatic resection: Where is the limit? *Surgery*, **111**, 251–259

Prof. Claude Huguet
Centre Hospitalier Princesse Grace
BP No 480
MC 98082 Monaco Cedex
Monaco

### TENSE ASCITES IN CIRRHOSIS: PARACENTESIS WITH ALBUMIN INFUSION VERSUS SPONTANEOUS ASCITES FILTRATION

### ABSTRACT

*Bruno, S., Borzio, M., Romagnoni, M., Battezzati, P. M., Rossi, S., Chiesa, A., Podda, M. (1992) Comparison of spontaneous ascites filtration and reinfusion with total paracentesis with intravenous albumin infusion in cirrhotic patients with tense ascites. BMJ*; **304**: 1655–1658.

